# Involving people with lived experience of homelessness in palliative and end of life care research: key considerations from experts in the field

**DOI:** 10.1186/s40900-024-00549-3

**Published:** 2024-01-30

**Authors:** Jodie Crooks, Kate Flemming, Caroline Shulman, Emma Casey, Briony Hudson

**Affiliations:** 1 Marie Curie, London, UK; 2 University of York, York, UK; 3 Pathway, London, UK; 4 Marie Curie Palliative Care Research Department, University College London, London, UK; 5 Groundswell, London, UK

**Keywords:** Palliative care, Homelessness, Inclusion health, Co-production, Patient and Public Involvement

## Abstract

**Background:**

Co-production of research aims to include people with lived experience of a phenomena throughout the research process. People experiencing homelessness often experience advance ill-health at a young age, yet access palliative care services at a disparately low rate to the level of palliative care need. The voices of people experiencing homelessness are infrequently heard throughout palliative care research, despite the complexities and intricacies of the area.

**Aim:**

To explore the experiences of experts in the field to identify key context considerations for involving people with lived experience of homelessness in palliative and end of life care research.

**Methods:**

Qualitative study comprising two data collection streams: interviews with professionals with experience of involving people experiencing homelessness in their work, and focus groups with people with lived experience (PWLE) of homelessness. Data were analysed using iterative, reflexive thematic analysis. Patient and Public Involvement contributors gave feedback on themes.

**Results:**

A total of 27 participants took part in semi-structured interviews (N = 16; professionals) or focus groups (N = 11; PWLE homelessness). Key considerations of involving people experiencing homelessness in palliative and end of life care research were developed into four key themes: complexity of lived experience of homelessness; representation of homelessness within experts by experience; professionalising lived experience; and methods for involvement.

**Conclusions:**

Involvement of people with lived experience of homelessness is important in developing palliative care research. This paper begins to outline some contextual considerations for promoting involvement in a complex and intricate field of research.

## Abstract (plain language summary)

People experiencing homelessness often become unwell at a young age. They often experience several illnesses at the same time, and many people experiencing homelessness may also experience substance misuse disorders and/or mental illness.

Despite this, they often are not identified as needing palliative care support, therefore rarely access services. Research into palliative care and homelessness may benefit from including people with lived experience of homelessness, yet this is rarely done, and is a sensitive and challenging area. The current study carried out interviews with professionals who have previously involved those with lived experience of homelessness in their work, and focus groups with people with lived experience of homelessness. Twenty seven participants took part: 16 professionals with extensive experience of supporting PEH and 11 people with lived experience. Key considerations of involving people experiencing homelessness in palliative care research were developed into four key themes: complexity of lived experience of homelessness; representation of homelessness within experts by experience; professionalising lived experience; and methods for involvement. Involvement of people with lived experience of homelessness is important in developing palliative care research. This paper begins to outline some contextual considerations for promoting involvement in a complex and intricate field of research.

## Introduction

### What is co-production?

Frequently seen as a logical evolution from traditional Patient and Public Involvement (PPI), co-production aims to move beyond simple consultation of people with lived experience (PWLE) [1]. Instead, co-production in research aims to involve the target audience for its intended outcomes in the conceptualisation, delivery and dissemination of research [1, 2]. Co-production prioritises enabling PWLE to “work safely outside the hierarchy” [3], through recognising the unique expertise and perspectives of those with lived experience. The ultimate aim is to integrate this knowledge in substantial and meaningful ways, to create impactful research informed by real-life experiences. This collaboration has also been referred to as ‘involvement’ by the National Institute for Health and Care Research (NIHR); “where members of the public are actively involved in research projects and research organisations” [4].

### Lived experience of homelessness

Lived experience has been defined as “the experience(s) of people on whom a social issue, or combination of issues, has had a direct impact” [5]. The lived experience of homelessness is a complex phenomenon, given the vast array of unique experiences across multiple types of homelessness [6]. Traditional perceptions of homelessness include street homelessness or rough sleeping, and temporary accommodation such as hostels and shelters [7]. However, homelessness can also exist within housing, where people are forced to live in unsafe or unstable environments, or housing that is unfit for habitation [8]. Lived experience of homelessness is increasingly complex when we consider the multiple forms of exclusion that this population may encounter. Multiple exclusion homelessness is defined as homelessness plus one of more other domain of social exclusion, including institutional care, substance misuse, or participation in ‘street culture activities’ [9].

As society, time and the environment progress, people’s experiences of a phenomena change: individual’s experiences of homelessness are likely to be impacted by societal fluctuations. For example, people experiencing homelessness at the time of writing are also faced with a cost of living crisis in the UK, that has contributed to increasing rent prices, eviction and first time homelessness [10]. People experiencing homelessness during the Covid-19 pandemic were particularly vulnerable, due to lack of safe space in which to isolate from the pandemic, and poor access to healthcare if they became unwell [11]. In recognition of this, during the Covid-19 pandemic, over 37,000 people experiencing homelessness were moved into temporary accommodation as part of the Everyone In campaign in the UK. This demonstrated the potential impact of political will in addressing homelessness [12]. Despite the vast range of experiences of people experiencing homelessness, all experience is valid and valuable for involvement in palliative care research.

### Palliative care and homelessness

#### Multimorbidity and premature death

Although there is a growing body of evidence around palliative care and homelessness in general, particularly the challenges in accessing palliative care faced by this group, research and service provision remains under-prioritised disparate to the high level of need.. Many people with multiple exclusion homelessness have advanced ill-health, often at a much younger age than the general population. Within this, there are high rates of multimorbidity of illness [13, 14]. Tri-morbidity, presence of a physical health condition, substance misuse and mental ill-heath, is disproportionately high in people experiencing homelessness [15]. A UK study explored the health needs of 2776 individuals currently experiencing homelessness. 82% of individuals had a mental health diagnosis, a quarter self-reported co-existing mental health and substance misuse needs, and a further 45% reported self-medicating with drugs or alcohol to help cope with poor mental health [16]. People experiencing homelessness also often experience accelerated ageing, geriatric conditions and premature frailty as early as in their 40’s or 50’s [17]. Consequently, relative to the general population, people experiencing homelessness often die at a young age [18].

#### Identification of palliative care need in people experiencing homelessness

The majority of PEH with advanced ill health are living in homeless hostels, rough sleeping, or experiencing hidden homelessness [19]. Many people experiencing homelessness are not diagnosed with a terminal disease until late in its trajectory, if at all, due to a range of reasons including barriers to health service access and unpredictable illness trajectories [19, 20]. Commonly used prognostic tools such as the ‘surprise question’ are not suitable for assessing palliative need in PEH as there is a higher risk of dying from accident, overdose or suicide as opposed to solely long-term conditions [19, 21]. Self-identification as needing palliative care support is extremely low in PEH: many PEH are less open to considering that they may be approaching the end of their life [24]. The majority of people with multiple exclusion homelessness have experienced significant trauma in their lives, often starting in childhood. They have often experienced significant bereavements and loss: in the UK, the estimated number of deaths among people experiencing homelessness has increased by 54% since 2013 [22]. In addition, substances are often used to self-medicate or self-soothe as a way of coping and dealing with trauma and bereavement. This is likely to contribute to difficulties in considering or reflecting on death and dying, self-identification of palliative care need, and accessing palliative care services.

#### Access to palliative care for people experiencing homelessness

People experiencing homelessness rarely have access to palliative and end of life care, despite the high level of need and symptom burden [19, 23]. Homelessness services are typically recovery-focussed in nature; they concentrate on supporting individuals to move towards independent living [24]. This contrasts the opposing dialogue of palliative care services, where recovery-based outcomes are often not possible and the focus is more about supporting people to live well, whatever that means for them, and for however long they may live. In addition to the often relatively young age of PEH with advance ill-health, these factors accentuate the dissonance in identifying young PEH as patients requiring palliative care. As a result, palliative care is often not considered as an option, or is deemed unsuitable for PEH. Consequently, many people experiencing homelessness with advanced illness do not receive the required support, leading to unsafe, undignified, often traumatic deaths.

#### Involvement of PWLE homelessness in PEoLC

Over recent years, some research has begun to focus on co-production of palliative care research [25–27], or involvement of PWLE homelessness in research generally [28, 29]. However, the critical overlap of these two fields is yet to be considered. A recent rapid review by this research team identified only three papers reflecting on co-production of palliative care research for inclusion health groups [30]. Within this, only one paper discussed involvement of PWLE homelessness [31].

#### Why do we need guidance for the involvement of PWLE homelessness in palliative and end of life care research?

People with lived experience of homelessness have often experienced trauma throughout their lives, often beginning at a young age or in childhood. This trauma is often complex, and impacts their experience of homelessness. In addition, PWLE homelessness have often experienced bereavements and been exposed to deaths, often of a traumatic nature such as suicide, overdose or accidents. The complexities of trauma and exposure to deaths for people experiencing homelessness contribute to sensitivity in discussing palliative care and dying. As a result, it is essential that any involvement in research of this topic is safe, supported and prioritises the wellbeing of the person: being trauma-informed is integral to this. Involvement of PWLE homelessness in palliative care research is a scarcely considered area in its infancy, and therefore requires in-depth exploration around the specific complexities of involvement with this population, in a potentially emotive topic.

### Aims

The aim of the current paper is to explore the experiences of experts in the field (health and social care professionals, researchers, and PWLE homelessness) to identify key context considerations for involving people with lived experience of homelessness in palliative and end of life care research.

## Methods

Data within the current study emerged as a strand of a larger qualitative study by the research team: full details on methodology, data collection and analysis is reported there (TIFFIN recommendations; in press).

### Participants and recruitment

Two main participant groups were recruited: professionals within the field and people with lived experience of homelessness. For professionals to be eligible for participation, they needed to have experience of co-producing or involving PWLE homelessness in their work, within the field of palliative care, whether this was research or service development. Any individual who self-defined as having previous or current experience of homelessness, who was able and willing to articulate their views around palliative care research was eligible for involvement.

Opportunistic sampling was chosen to recruit professionals via existing networks of the research team and identifying authors and team members of published literature or works.

People with lived experience of homelessness were recruited through Groundswell, a third sector peer advocacy organisation. Eligible participants were those who had experience of being involved in health research, or who were interested in palliative care research involvement. They were approached and recruited via opportunistic sampling, by an experienced Peer Coordinator employed by Groundswell, who had an existing relationship with potential participants.

### Ethical considerations and researcher positionality

Ethical approval was sought and obtained from University College London (approval ID: 6202/008). The expertise of Groundswell’s Peer Coordinator, who had pre-existing rapport with potential participants, guided recruitment. To avoid the small potential for coercion, participants were given a minimum of 24-h for consideration of participation, and discussed this with others. Verbal consent was sought at the beginning of each focus group with people with lived experience of homelessness, by the Peer Coordinator overseen by a member of the core research team.

Members of the core research team (BH, CS) have experience in carrying out qualitative research with participants currently experiencing, and with previous lived experience of homelessness. CS is also an inclusion health clinician, who provided a clinical viewpoint and mentorship. Their experience and expertise within this informed the design of the research, and identified potential areas for ethical concern early in the planning stage of the research. Other research team members (JC, KF) have significant experience in qualitative research methodologies, specifically within topics of inclusion health and social justice issues.

### Data collection

Data were collected between January 2023 and June 2023. Professionals partook in semi-structured interviews discussing their experiences of involving PWLE homelessness in their palliative and end of life care work. Interviews were carried out online, via MS Teams. A semi-structured approach was chosen to allow for flexibility in discussion, and encourage participants to discuss issues salient to their experience.

People with lived experience of homelessness were invited to attend one of two focus groups. These stimulated discussions around any experiences of being involved in research (as a co-researcher), thoughts around barriers and facilitators to involvement, and advice for researchers hoping to involve PWLE homelessness in their palliative care research. Recruitment, set up and delivery of focus groups were supported by an experienced Peer Coordinator employed by Groundswell (EC). Groundswell are a third sector peer-advocacy organisation, with whom the research team have ongoing, working relationships. Focus groups were carried out online via MS Teams, lasting 90-min. All interviews and focus groups were audio recorded and transcribed verbatim.

### Data analysis

Reflexive thematic analysis was used to develop themes and in turn recommendations from the data collected. This analysis method was chosen as it “emphasises the importance of the researcher’s subjectivity as analytic resource, and their reflexive engagement with theory, data and interpretation” [32]. Given that the core research team are experienced researchers in the field of palliative care and homelessness, and advocate for greater, meaningful involvement of PEH in palliative care research, being aware of and respecting their subjectivity through reflexive thematic analysis allowed for production of data-based themes that were tied to the researchers experiences.

The six proposed steps for reflexive thematic analysis were worked through by two members of the team (JC, BH) [32]. After familiarisation with the data, line by line coding was carried out to produce a set of initial codes. These were then constructed into initial themes, which were shared back to participants (professionals and PWLE homelessness) to gather feedback and encourage iterative development of the themes. This led to interpretative themes generated through discussion with identified end-users of the research, and the wider research team.

### Patient and public involvement

Patient and Public Involvement was gathered in two main ways. First, an additional 5 people with lived experience of homelessness were recruited through Groundswell. They gave advice and feedback on the themes and recommendations developed through the study. Second, a member of the core research team (EC) has lived experience of homelessness. They were involved in data collection, analysis, and development of the manuscript.

## Results

A total of 27 participants took part in semi-structured interviews (N = 16; professionals) or focus groups (N = 11; PWLE homelessness). Four key considerations around involving people with lives experience of homelessness were identified: complexity of lived experience of homelessness; representation of homelessness within experts by experience; professionalising lived experience; and methods for involvement.

### Complexity of lived experience of homelessness

Participants emphasised the complex reality of homelessness in modern day, and the need to move beyond traditional interpretations of homelessness focused on rough sleeping and hostel accommodation when recruiting people with lived experience to take part in palliative and end of life care research.

We were conscious that if you use the term homelessness, it means different things to different people. For some people it is literally people who live in a sleeping bag on the streets, or for some people, they think it’s hostels and only that. (Hospice Nurse)

This diversity should also be considered in regard to lived experience of ill-health, advance illness and bereavement, with participants suggesting there are *“different dimensions” (Professor)* to lived experience of palliative care and homelessness. Therefore, a person with lived experience of homelessness who joins a research team as a co-producer within a palliative care project, could have a diverse range of complex experiences sitting within a vastly heterogeneous range, which may differ significantly from others in the same locations.

One person doesn’t replace another person in my experience. Like multiple different perspectives, multiple different experiences, intersectionality needs to come into play. And even within certain social locations there's going to be differences (Researcher)

For this reason, participants reported rarely using a formal definition of homelessness for eligibility criteria of lived experience, deeming it too reductionist for this complex experience. Instead, participants allowed people to assign their own ‘labels’ to their experience, as opposed to assuming or othering based on predetermined definitions of homelessness.

We’re never going to disregard somebody’s homelessness and homelessness experience or, you know, create a kind of hierarchy of experiences. (Peer Coordinator)

However, difficulties sometimes arose where individuals did not explicitly or openly identify as having experienced homelessness. Participants recognised that self-definition of lived experience can be complex, as it forces people to negotiate externally imposed labels of homelessness and the accompanying stigma.

Furthermore, participants reflected that experiences of a phenomena can be both past and present, manifesting both living and lived experience. It is likely that individuals involved in research may sit along a spectrum of homelessness experiences.

I think that’s where you find a pool of people that have got lived experience, like they’ve experienced homelessness like say 20 years ago. That’s lived experience. People who are currently homeless, that’s lived experience. People that, you know, the whole spectrum. (Palliative Care Professional)

### Representation of homelessness within experts by experience

Professionals reflected that it was common when involving people in research, to select people who ‘present well’.

We often select people that will fit in our narrative. It’s like a dominant narrative or something. (Homelessness Coordinator)

In recognising the emotional sensitivity in palliative care as a topic, participants discussed a degree of capability bias in selecting individuals to be involved. Professionals reported being aware of their own and others tendency to select individuals who hold particular skills or traits that may make them better equipped to engage in research involvement, or avoid those who do not. Though this selective practice may be seen as ‘othering’, participants expressed that it can be difficult to safely represent people living in present-tense, chaotic or traumatic positions.

Something that one needs to be careful of, around co-production and around any kind of research with homeless people, is to try and minimise the kind of capability bias. That you end up talking to and working with the people who are closest to you, in terms of what they’re mental state is, what they’re stability is, you know. (Professor of Social Work)

People with lived experience of homelessness illustrated that in order to feel able to be involved in research, they had to have experienced some degree of recovery.

I know when I first became homeless I probably wouldn’t have been able to volunteer anywhere because I just had so many day-to-day issues and I was trying to deal with being made homeless and I think that’s probably not an uncommon thing for many people. But later on when you're either in a better place or your mind is, you know, your mental health is better or whatever it is, it could be physical health is better, then you may be in a better place (PWLE homelessness).

### Professionalising lived experience

Both professionals and PWLE homelessness discussed the ‘spectrum’ on which an individual’s experiences may lie: with each period of research involvement, an individual’s experience becomes yet more layered. That is, people may begin to have both lived experience of the phenomena under research, and lived experience of carrying out research, policy or practice work.

#### "You know if you’ve been on the street for five years, but then you spent 20 years as a committee member, the 20 years is going to count for something as well." (PWLE homelessness)

Participants suggested that this can create a group of PWLE homelessness*,* who *“transition from lived experience to becoming consultants about lived experience*” *(Professor)*. This professionalisation of people’s experiences may become problematic, however, if they are expected or pressured to ‘use’ their lived experience to support research and help others; this pressure of ‘using experience for good’ could make people feel exploited.

I don’t think that there's like any clause that says now if you have experiences of this you must use them wisely…But it makes me sort of annoyed, you know, like it’s kind of, can you look sadder? Underneath that tree while I take a photo (Project Coordinator).

Participants discussed how this overlap of lived experiences may create a range of ‘pure lived experience’ to ‘pure academic research experience’, that should be considered throughout research projects. Importantly, however, participants emphasised that full-time staff employed by institutions into research posts, could also have undisclosed lived experience of a related circumstance; people are unlikely to fall exclusively into any specified category.

Because the next thing is, this is where the discourse of lived experience gets quite tricky, because if we start trying to sort of put boundaries around what people can and can’t do, it’s also potentially othering because, you know, I’m a Lecturer at (Institution). I might also have been homeless in my previous life, but nobody would badge me as having lived experience. (Senior Lecturer).

### Methods for involvement

Participants (both professionals and PWLE homelessness) reported involvement in a range of research activities. A distinction was drawn between legitimate involvement in research (i.e., co-research), versus simple consultation. Examples of areas of involvement included collaboration on research question formulation, being co-applicants on research grants, data collection (such as co-interviewing), involvement in policy recommendation development, and feedback on services to aid further development. A number of professionals reported encouraging individuals with lived experience to lead parts of the project, and allowing flexibility and creativity in the research process to foster true co-production.

*“From my perspective getting as far out the way as possible while still keeping a kind of eye on things, is where we position it. I mean you’re not being co-productive if you’re micromanaging…You know if you’re saying, “Well, you’re a person with lived experience, but you must do this, and you should ask these questions, and you shouldn’t ask them anything else.” Then you know you have to think about giving them some creative leeway.” (Professor of Social Work).*

A critical facet of involvement reported by professionals was involving people with lived experience as early in the process as possible, to avoid mistakes or oversights in the design of the research, and allow researchers to see the topic of research from a different perspective. This was also reflected by PWLE homelessness, who appreciated the opportunity for early involvement, and recognised past involvement where they felt they were introduced ‘too late’.

I've been involved in things before where they’re [researchers] like, oh, we’ve got it all together, it’s all sorted but we just want your input and then you give the input and they’re like, oh, we’ve made a mistake, we’ve done this wrong. We should have involved you at the beginning. Because they’ve looked at the wrong things. They need to talk to people who’ve actually experienced it to get the right picture. (Person with lived experience of homelessness)

## Discussion

The current study reports key considerations by experts in the field for involving PWLE homelessness in palliative and end of life care research. Data from sixteen professionals and eleven PWLE homelessness developed into four key themes: complexity of lived experience, representation of homelessness within experts by experience; professionalising lived experience; and methods for involvement. This report is the first of its kind to illustrate the context surrounding and key considerations for co-production of palliative care research with PWLE homelessness. Subsequent to this paper, the authors of this paper have developed recommendations for involving PWLE homelessness in palliative care research (TIFFIN recommendations; in press). Although this research was carried out in the UK, with themes developed based on the context of UK homelessness, the learnings are likely to be important to other countries, too.

### Self-defining lived experience of homelessness

Previous literature has explored the complexities of self-identifying as having lived experience of homelessness against societal prejudices. One study with young people experiencing homelessness illustrated that the negative social perceptions of being homeless caused challenges in identity development [33]. Consequently, when people experiencing homelessness construct their identity, they likely confront stigma and prejudice, making it difficult to openly self-define as having experienced homelessness. Evidence has suggested that this can be applied to their involvement as lived experience co-researchers. In explicitly allowing their lived experience to be a visible, core part of involvement, PWLE homelessness may fear that their credibility as researchers and the knowledge they contribute may be doubted [34]. This may be in part explained by social identity theory: even after self-defining that lived experience of homelessness is a part of their identity, this may be seen as the “in-group” to which they belong, with academic researchers forming an”out-group” [35]. The fear of stigma even within the “safe hierarchy” as co-production aims to form can be complicated for PWLE homelessness, and impact their willingness to share parts of their identity.

### The need for involvement

In addition to demonstrating key considerations for co-producing palliative care research with PWLE homelessness, the current study illustrates the need for involvement. Researchers alone cannot dictate the gaps and priorities of research without the input of individuals with lived experience; perspective from the people for whom the work is intended to benefit is key. Involvement and co-production ensure that lay people have a voice in shaping work that affects them directly, creating respectful relationships across societal hierarchies [36]. The experience of homelessness in combination with palliative care is an exceedingly complex phenomenon that when standing alone, researchers can only begin to explore.

Furthermore, involvement gives legitimacy to many areas of the research process. For academic researchers, it helps them to gain ‘experiential knowledge’ and begin to understand experiences of homelessness through proxy exposure to people’s reality [37]. The complexity of lived experience illustrated in this study demonstrates the necessity for researchers to have some understanding of the lived experience of homelessness prior to commencing involvement, as naivety and lack of preparedness for involvement has the potential to be dangerous or harmful for all. Involvement of individuals with lived experience has also been shown to increase recruitment and follow-up rates, add to the validation of findings and generate more useful outputs [38].

### Involvement in palliative care research

People experiencing homelessness often experience advance ill-health at a young age, alongside accelerated ageing and frailty [13]. This often leads to multi- or tri-morbidity of physical ill-health, mental ill-health and substance misuse [14, 16]. Due to a number of factors such as unpredictable illness trajectories, palliative care need remains under-identified in people experiencing homelessness. This can result in difficulties accessing services, and people dying without the support they need.

These challenges surrounding palliative care for people experiencing homelessness are confronted further by the trauma frequently experienced by these individuals. High levels of bereavement and exposure to often traumatic deaths can mean that future discussions around death, dying and palliative care need to be handled with utmost sensitivity and care.

Given the complexity of the field of palliative care and homelessness, it is essential to include the voices of PWLE homelessness in research. It is key to revealing the ‘unknown unknowns’ to academic researchers around the complexities and intricacies of palliative care and homelessness. When done safely and genuinely, involvement can direct us to researching ‘what is right’, including the voices of a frequently unheard group, to create positive, impactful change.

### Culture shift around involvement

Within the research landscape, there has been a shift in culture away from researcher-led work, towards higher levels of involvement of those with lived experience. Whereas previously co-production may have only been done by those who were passionate about the process, co-production, co-research or Patient and Public Involvement in some format is often now expected as part of a research funding process [39]. Although this shift is largely perceived as positive, there are concerns that compulsory involvement may lead to involvement becoming tokenistic. This has been described as a “semantic rather than substantive shift” [40], whereby co-production or involvement become buzzwords used by researchers to secure funding and appease committees. Particularly in the field of palliative care and homelessness, the risk of exploitative involvement against a structurally vulnerable population is cause for concern.

### Patient and public involvement

Input from people with lived experience of homelessness was key throughout the development of these themes, and corresponding recommendations (TIFFIN recommendations, in press). Feedback was gained on the initial themes, which affirmed the content and highlighted areas for clarification. For example, PPI input contributed to the theme of ‘defining lived experience of homelessness’: they highlighted the complexities and intricacies rooted in their real life experiences. In addition, one core member of the team has lived experience of homelessness. They were integral to the recruitment process of other PPI representatives, and PWLE homelessness to participate in focus groups. They were able to utilise their existing relationships to engage with and recruit participants, and facilitate focus groups.

### Limitations

Recruitment of PWLE homelessness was purposeful and prioritised safe participation over quantity of participants. Although this approach is preferred, additional time and resource to allow meaningful recruitment of more participants with lived experience may have expanded the range of experiences and viewpoints included. Additionally, as PWLE homelessness were approached by Groundswell’s staff, it is unknown how or whether those who declined to participate differ from those that took part and the extent to which gatekeeping impacted recruitment.

## Conclusion

Involving people experiencing homelessness in palliative and end of life care research is important to give legitimacy to research into a complex range of heterogeneous experiences. When referring to lived experience of homelessness, it is important to be aware of the broad range of unique and complex experiences that this encompasses. Palliative care in the context of homelessness can illustrate further complexities within individual’s experience. While co-research practice is increasing, care must be taken to avoid tokenism or exploitative practices for involving people experiencing homelessness in palliative and end of life care research. Keeping the best interests of the individual at the centre of our work, above and beyond following traditional research processes, is critical to promote ethical, safe and considered involvement for this population. Our best practice guidance and recommendations (in press) can support researchers to engage in co-production of palliative care research with people with lived experience of homelessness.

## Data Availability

The datasets used and/or analysed during the current study are available from the corresponding author on reasonable request.
